# Successful staged anatomic repair for congenitally corrected transposition with both atrioventricular valves’ regurgitation: a case report

**DOI:** 10.1186/s44215-026-00281-2

**Published:** 2026-10-05

**Authors:** Yuji Fuchigami, Koichi Toda, Akinori Hirano, Ryusuke Hosoda, Yukino Iijima, Takaya Hoashi

**Affiliations:** 1 Department of Pediatric Cardiac Surgery, Saitama Medical University International Medical Center, 1397-1, Yamane, Hidaka, Saitama 350-1298 Japan; 2 Department of Pediatric Cardiology, Saitama Medical University International Medical Center, 1397-1, Yamane, Hidaka, Saitama 350-1298 Japan

**Keywords:** Congenitally corrected transposition of the great arteries, Congenital mitral regurgitation, Anatomical repair

## Abstract

A 4-year-old boy with dextrocardia, congenitally corrected transposition of the great arteries, pulmonary valve stenosis, and ventricular septal defect demonstrated progressive hypoxemia and mild-to-moderate mitral and tricuspid regurgitation. Cardiac magnetic resonance imaging revealed insufficient right ventricular volume for Senning-Rastelli type anatomical repair. Given the elevated surgical risk associated with mitral regurgitation and inadequate ventricular volume, an initial palliative approach was undertaken at 2 years of age, consisting of systemic-to-pulmonary shunt placement and mitral valve repair, which improved oxygenation and promoted right ventricular growth, with trivial mitral regurgitation. Subsequent imaging confirmed adequate ventricular development and trivial mitral regurgitation, allowing for definitive anatomic repair at 4 years and 7 months of age. The procedure comprised a Senning atrial switch combined with a Rastelli operation. The postoperative course was uneventful, and imaging demonstrated preserved biventricular function with minimal atrioventricular valve regurgitation and no evidence of outflow or pathway obstruction.

## Background

Whereas existence of significant tricuspid regurgitation (TR) represents fragility of right ventricle therefore anatomic repair is justified for congenitally corrected transposition of the great arteries (ccTGA) [1–10], concomitant significant mitral regurgitation (MR) makes decision of definitive repair difficult. Herein we reported a case of ccTGA complicated by both TR and MR, who was successfully managed with a staged anatomical repair.

## Case presentation

The patient was a 4-year-old boy. Although fetal echocardiography suggested congenital heart disease, no definitive abnormalities were identified during the perinatal period, and he was delivered vaginally at 37 weeks and 6 days of gestation. Postnatal echocardiography revealed dextrocardia, ccTGA, pulmonary valve stenosis (vPS), and ventricular septal defect (VSD). Despite these findings, he remained hemodynamically stable without signs of heart failure and was discharged home, with subsequent follow-up consisting of regular outpatient visits and periodic hospital evaluations.

**Fig. 1:**
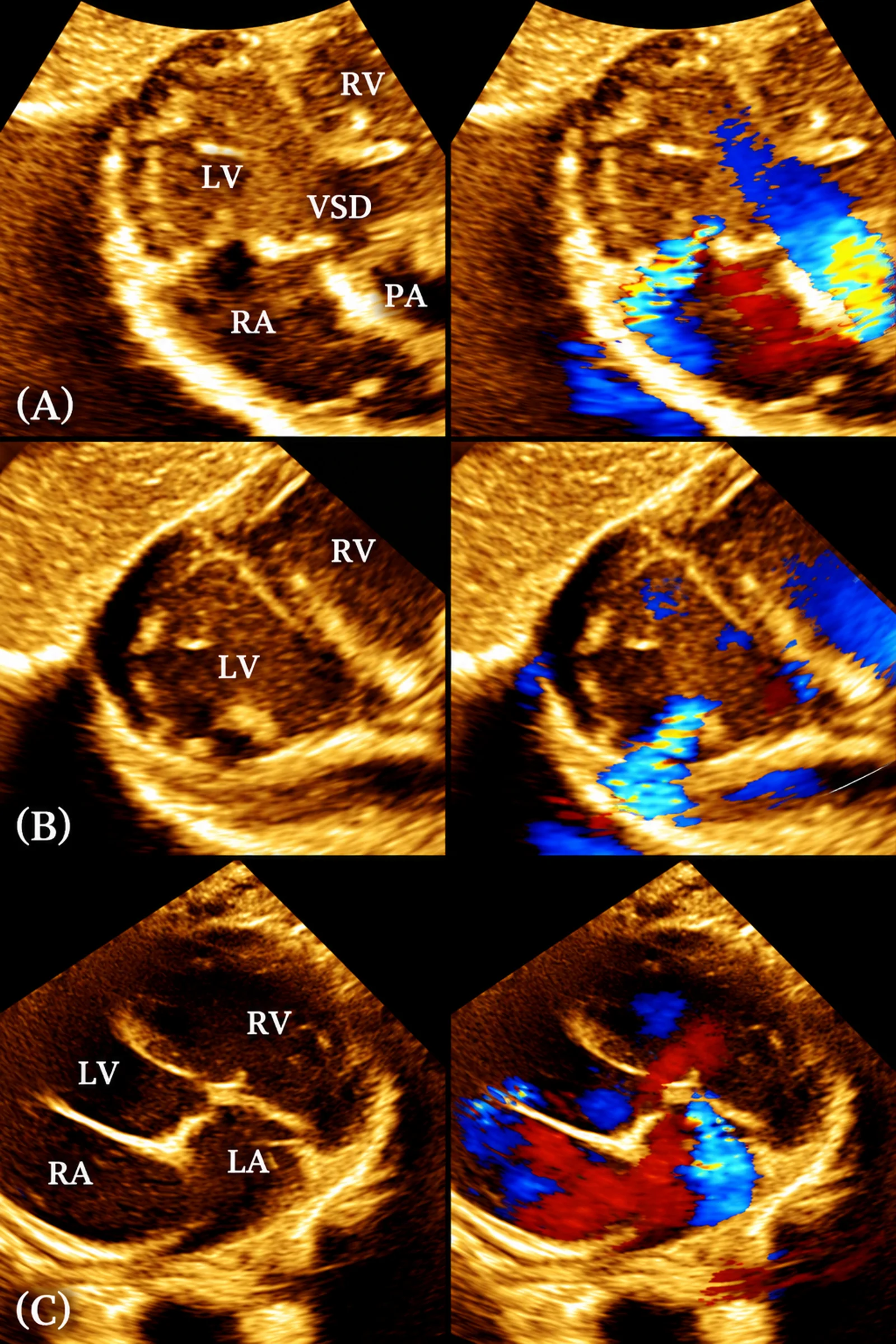
Preoperative transthoracic echocardiography. **A** Long-axis view showing mitral regurgitation (MR). **B** Short-axis view showing MR. **C** Long-axis view showing tricuspid regurgitation. LA, left atrium; LV, left ventricle; PA, pulmonary artery; RA, right atrium; RV, right ventricle; VSD, ventricular septal defect

At 1 month of age, Saturation of percutaneous oxygen (SpO₂) was 96%, but it progressively declined to 85% by 2 years of age, prompting consideration of surgical intervention. Echocardiography demonstrated mild-to-moderate TR as well as mild-to-moderate MR (Fig. 1). Cardiac magnetic resonance imaging (MRI) at 18 months showed a left ventricular end-diastolic volume (LVEDV) of 23.9 mL (113% of normal) and a right ventricular end-diastolic volume (RVEDV) of 29.4 mL (118% of normal). Although anatomical repair was deemed preferable in the presence of vPS and TR, the right ventricular volume was considered insufficient for such an approach. Furthermore, the coexistence of mild-to-moderate MR was regarded as an additional risk factor. Therefore, an initial palliative strategy involving shunt placement and mitral valve repair was selected to improve oxygenation and promote right ventricular growth, with the intention of performing definitive anatomical repair at a later stage.

The initial operation was performed at 2 years and 2 months of age, during which a 4-mm graft was placed from the innominate artery to the right pulmonary artery. Under cardiopulmonary bypass, inspection of the mitral valve revealed an indentation between segments A1 and A2 with leaflet thickening, which was repaired by direct closure.

Following surgery, SpO₂ improved to approximately 90%. At 3 years of age, cardiac MRI demonstrated LVEDV of 42.8 mL (123% of normal) and RVEDV of 55.3 mL (138% of normal), confirming adequate growth of the anatomical right ventricle. Echocardiography showed trivial MR, and the patient was therefore scheduled for anatomical repair as initially planned.

**Fig. 2:**
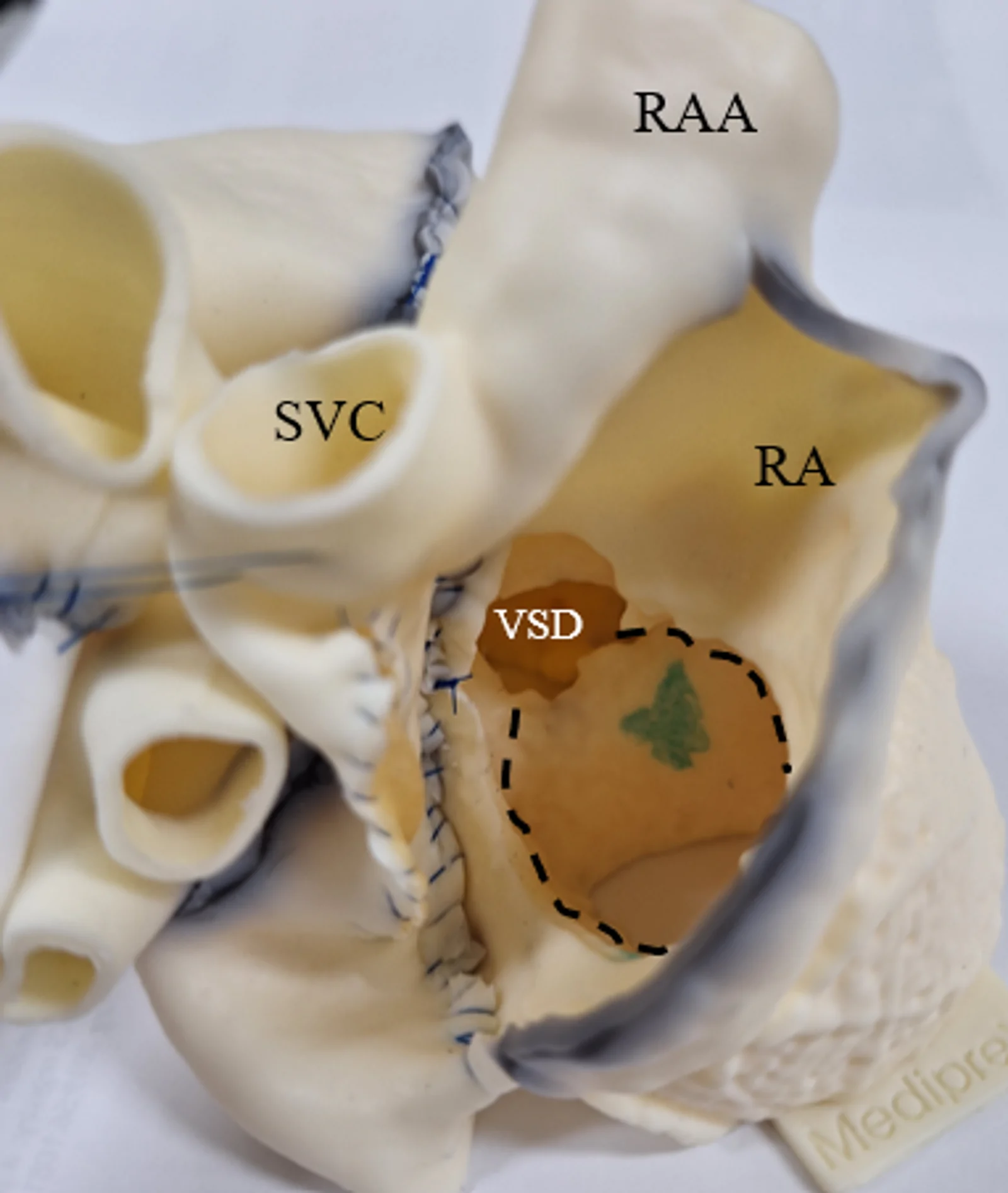
3D model of patient’s heart before anatomical repair. SVC, superior vena cava: RAA: right atrial appendage; RA, right atrium; VSD, ventricular septal defect; black dot line; annulus of mitral valve

At 4 years and 7 months of age, anatomical repair was performed using an atrial switch (Senning procedure) combined with a Rastelli operation. During the atrial switch, patches were utilized to augment the atrial septal and right atrial flaps. Due to inadequate extension of the main pulmonary artery root, a 6-mm graft was interposed during the Damus–Kaye–Stansel anastomosis. The subaortic VSD measured 14 mm, whereas the aortic annulus measured 22 mm, resulting in a VSD-to-aortic annulus diameter ratio of 0.64. Preoperative echocardiography and 3D modeling suggested that the VSD was restrictive for intraventricular rerouting, raising concern for systemic outflow obstruction after intraventricular rerouting (Fig. 2). Therefore, a Damus–Kaye–Stansel anastomosis was added, and the VSD was closed using a 30-mm circular patch fashioned from a 14-mm graft to achieve intraventricular rerouting.

**Fig. 3:**
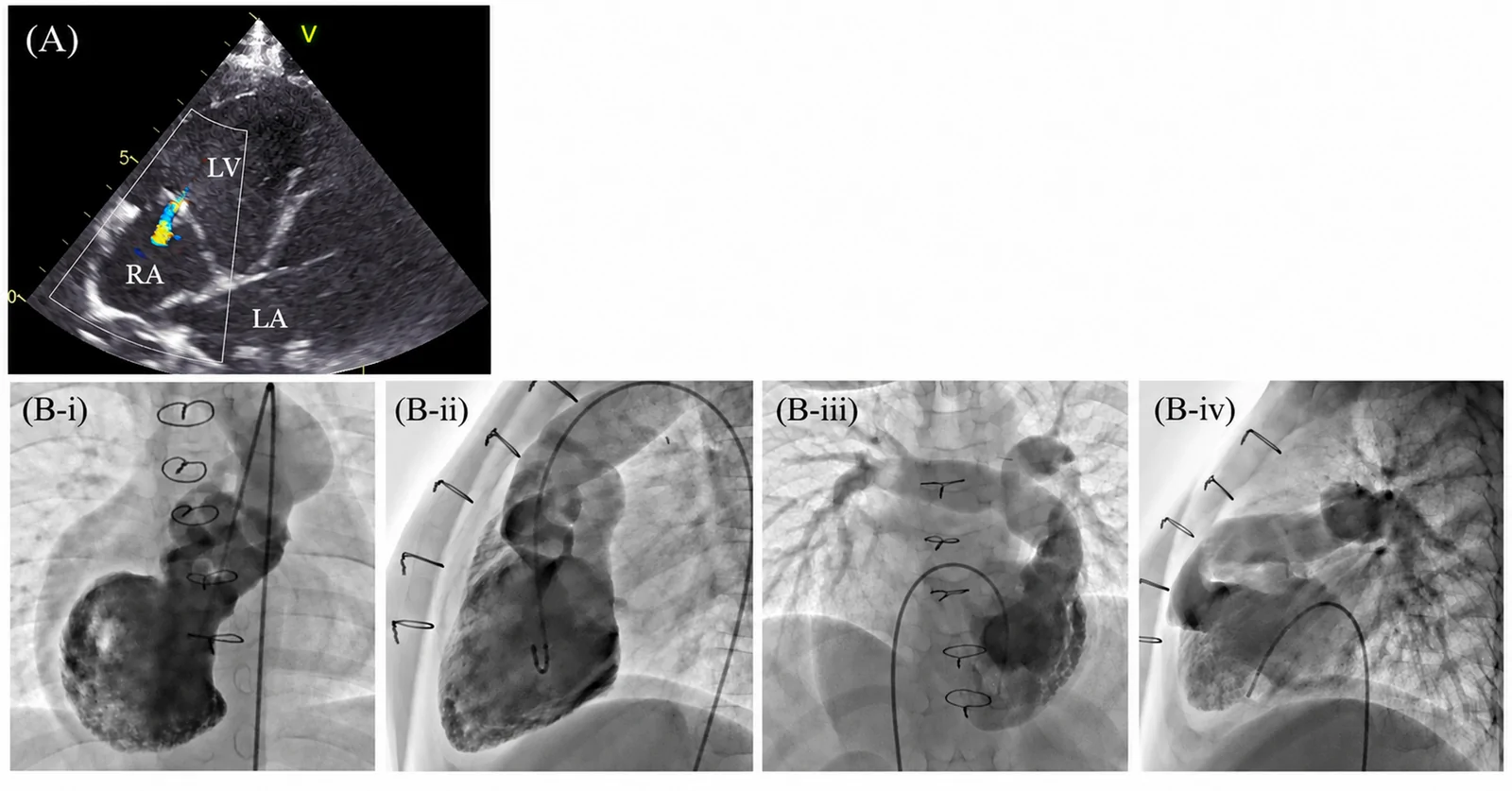
Postoperative imaging findings after anatomical repair. **A** Long-axis view demonstrating trivial mitral regurgitation on transthoracic echocardiography. **B** Cardiac catheterization. (ⅰ) Frontal view of left ventriculography. (ⅱ) Lateral view of left ventriculography. (ⅲ) Frontal view of right ventriculography. (ⅳ) Lateral view of right ventriculography. LA, left atrium; LV, left ventricle; RA, right atrium

Postoperative echocardiography demonstrated minimal MR and TR, while CT confirmed the absence of outflow tract obstruction or caval pathway obstruction. The patient was discharged home on POD 36 and continued to be followed in the outpatient clinic. At the 8-month postoperative cardiac catheterization, both MR and TR remained trivial, and no stenosis of the Senning pathway was identified. The left ventricular outflow tract pressure gradient was approximately 15 mmHg, and the left ventricular end-diastolic pressure was 11 mmHg, indicating preserved ventricular function (Fig. 3).

## Discussion

Reported patients with ccTGA and MR who underwent anatomic repair were limited (Table 1). Among the published literature, only 12 patients were reported to have MR before anatomical repair, and only one patient underwent mitral valve intervention before definitive repair. However, the details of these cases were not described, and it remains unclear whether the MR was congenital in origin or developed secondarily following palliative procedures, such as pulmonary artery banding or a systemic-to-pulmonary shunt. This likely reflects both the rarity of congenital MR in patients with ccTGA and the infrequent selection of anatomical repair in such patients. In contrast, 17 patients have been reported to undergo concomitant mitral valve intervention at the time of anatomical repair. It is conceivable that, in many of these patients, MR was initially mild and subsequently progressed during left ventricular training with pulmonary artery banding, resulting in a combination of anatomical and functional MR.

**Table 1:** Mitral regurgitation and mitral interventions related to anatomical repair of ccTGA and related diseases

	Publication year	Authors	Number of cases	Procedures of AR	Preoperative MR (%)	Preoperative MV intervention (%)	Concomitant MV intervention (%)
1	2007	Shin’oka et al.	84	DS 15, ASR 69	7 (8)	-	7 (8)
2	2009	M.Ly et al.	20	DS 20	-	-	-
3	2009	Gaies et al.	65	DS 35, ASR 30	-	-	-
4	2011	Murtuza et al.	113	DS 68, ASR 45	-	-	1 (1)
5	2011	Malhotra et al.	48	DS 23, ASR 25	-	-	-
6	2011	V. Hraska et al.	23	DS 10, ASR 13	-	-	-
7	2013	Hoashi et al.	47	ASR 47	-	-	2 (4)
8	2020	K. Ma et al.	85	DS 34, ASR 51			4 (5)
9	2022	Marathe et al.	103	DS 103	2 (2)	1 (1)	3 (3)
10	2024	Yokohama et al.	29	DS 15, ASR 14	3 (10)	-	-

*AR* anatomical repair, *DS* double switch procedure, *ASR* atrial switch and Rastelli, -: No description

In the present case, the coexistence of mild-to-moderate TR and vPS favored anatomical repair consisting of an atrial switch combined with a Rastelli procedure. Although the long-term superiority of the atrial switch plus Rastelli procedure over physiological repair has been questioned, unlike that of the atrial and arterial switch procedure, previous studies have suggested that anatomical repair should be preferred when physiological repair would require VSD closure with placement of a left ventricle-to-pulmonary artery conduit [11]. Nevertheless, there were concerns that congenital MR might compromise postoperative systemic left ventricular function. Furthermore, intraventricular rerouting inevitably reduces the functional volume of the anatomical right ventricle, and a preoperative right ventricular volume of approximately 120% of normal has been considered necessary for successful anatomical repair [7]. In our patient, presence of TR raised concerns regarding the long-term reserve of the systemic right ventricle if physiological repair were selected. Because the morphological left ventricle would become the systemic ventricle after anatomical repair, we considered it important to repair the mitral valve and confirm adequate mitral valve competence and preserved left ventricular function before committing the patient to definitive repair. Therefore, mitral valve repair was performed at the initial palliative stage rather than concomitantly with anatomical repair. At the same time, progressive hypoxemia necessitated early surgical intervention, and a systemic-to-pulmonary shunt was placed to improve oxygenation and promote right ventricular growth through volume loading. This staged strategy allowed us to confirm adequate control of MR and sufficient biventricular development before proceeding to definitive anatomical repair.

Intraoperatively, the MR was found to be caused by a congenital indentation between the A1 and A2 scallops of the anterior mitral leaflet and was successfully corrected by direct closure. As a result, staged anatomical repair could be completed as planned. Although long-term follow-up remains necessary, both biventricular function and atrioventricular valve function remain well preserved at the latest follow-up.

## Conclusion

We report a case of staged anatomic repair for ccTGA with significant TR and MR who successfully underwent Senning-Rastelli type anatomic repair following mitral valve repair and placement of systemic to pulmonary shunt. Serial follow-up of mitral valve function is warranted.

## Data Availability

No additional data to disclose.

## Abbreviations

ccTGA: Congenitally corrected transposition of the great arteries
LVEDV: Left ventricular end-diastolic volume
MR: Mitral regurgitation
MRI: Magnetic resonance imaging
POD: Postoperative day
RVEDV: Right ventricular end-diastolic volume
SpO2: Saturation of percutaneous oxygen
TR: Tricuspid regurgitation
VSD: Ventricular septal defect
vPS: Pulmonary valve stenosis
